# Supplementary figures and images for: A nucleation barrier spring-loads the CBM signalosome for binary activation
Source: eLife. 2022 Jun 21;11:e79826. doi: 10.7554/eLife.79826 (PMC9342958; doi:10.7554/eLife.79826)

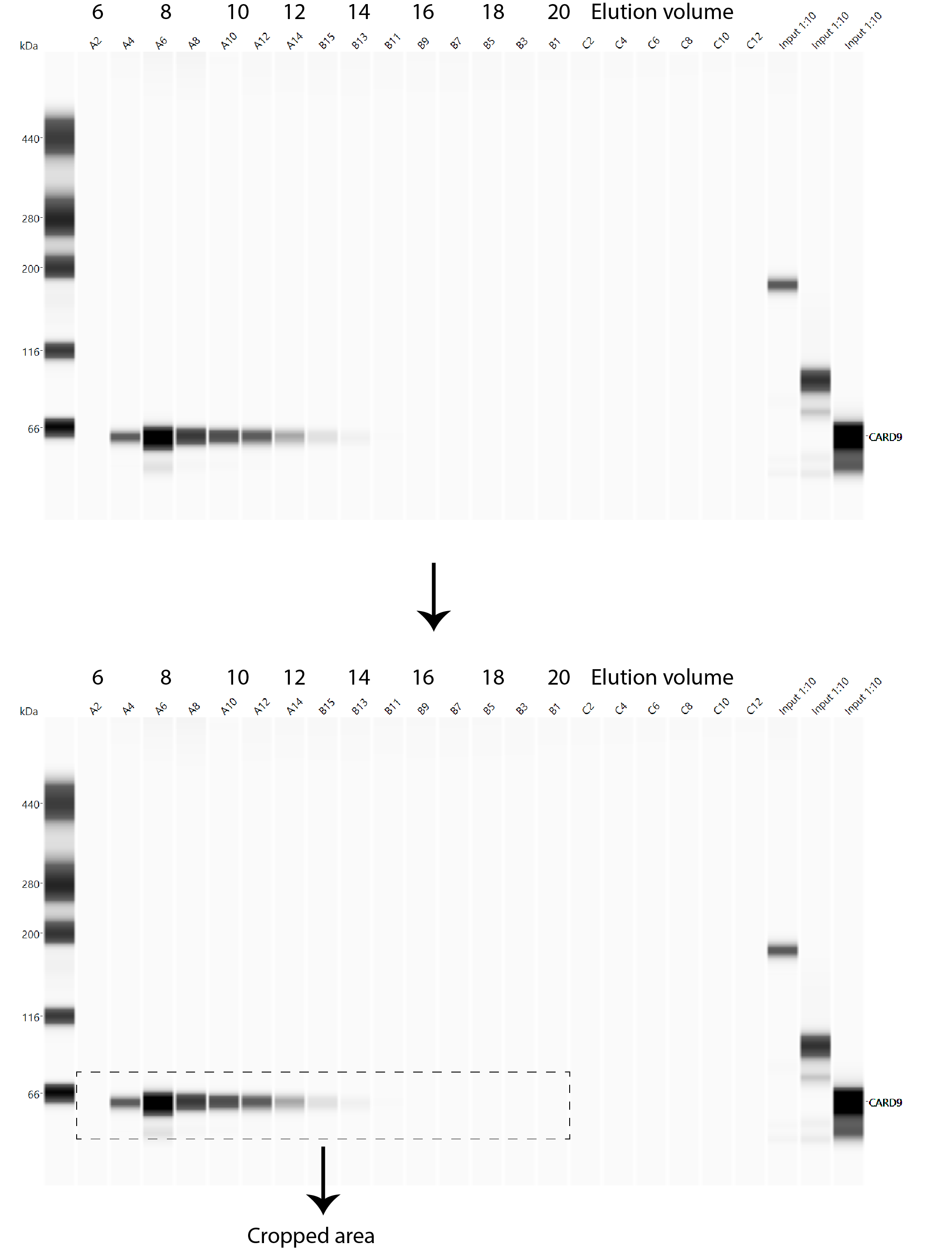

Supplement: Figure 1—source data 1. [file elife-79826-fig1-data1.zip › Figure1-source data 1.png]

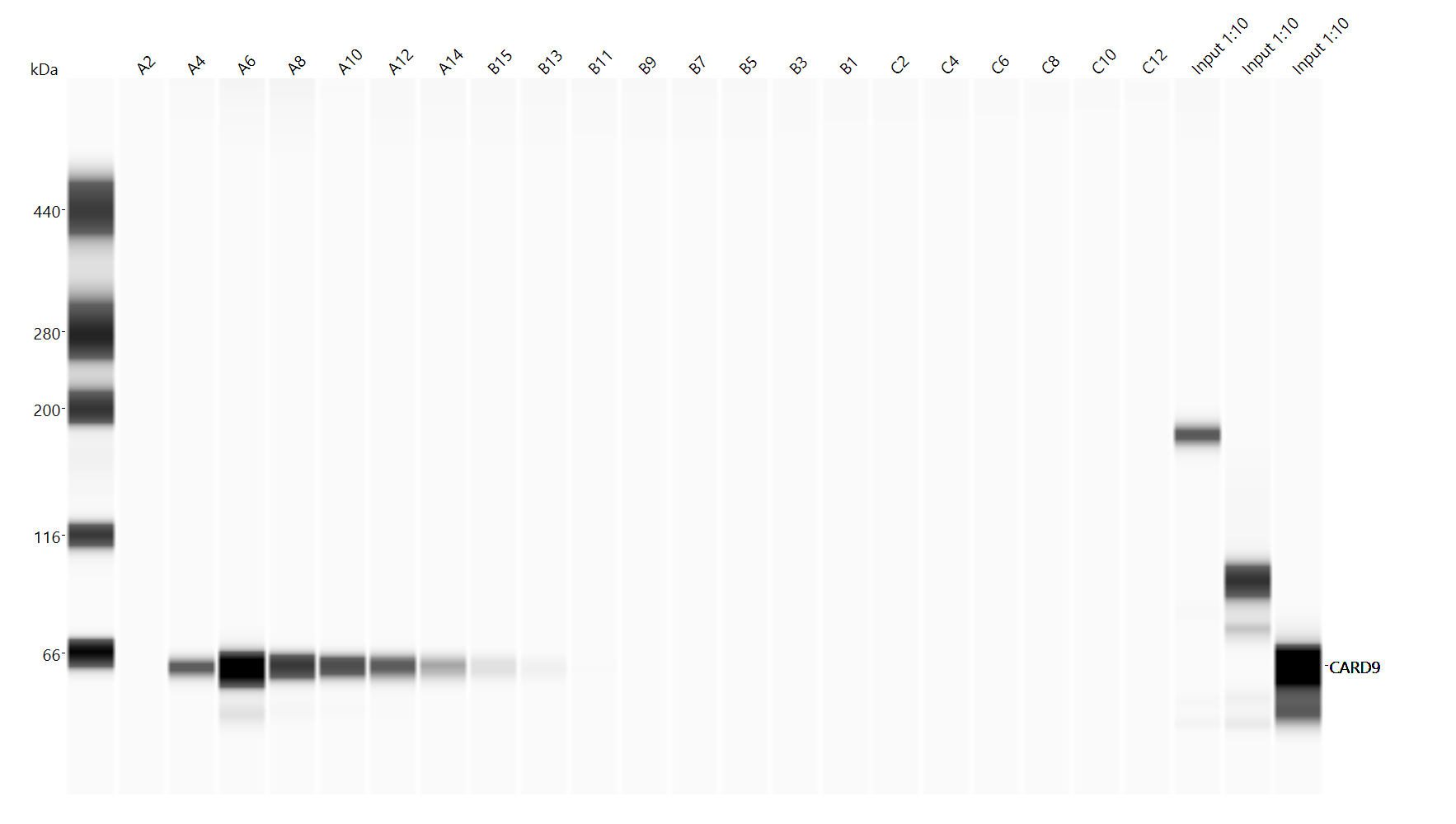

Supplement: Figure 1—source data 1. [file elife-79826-fig1-data1.zip › Figure1-source data 1_raw SEC_THP1_BGP_CARD9.png]

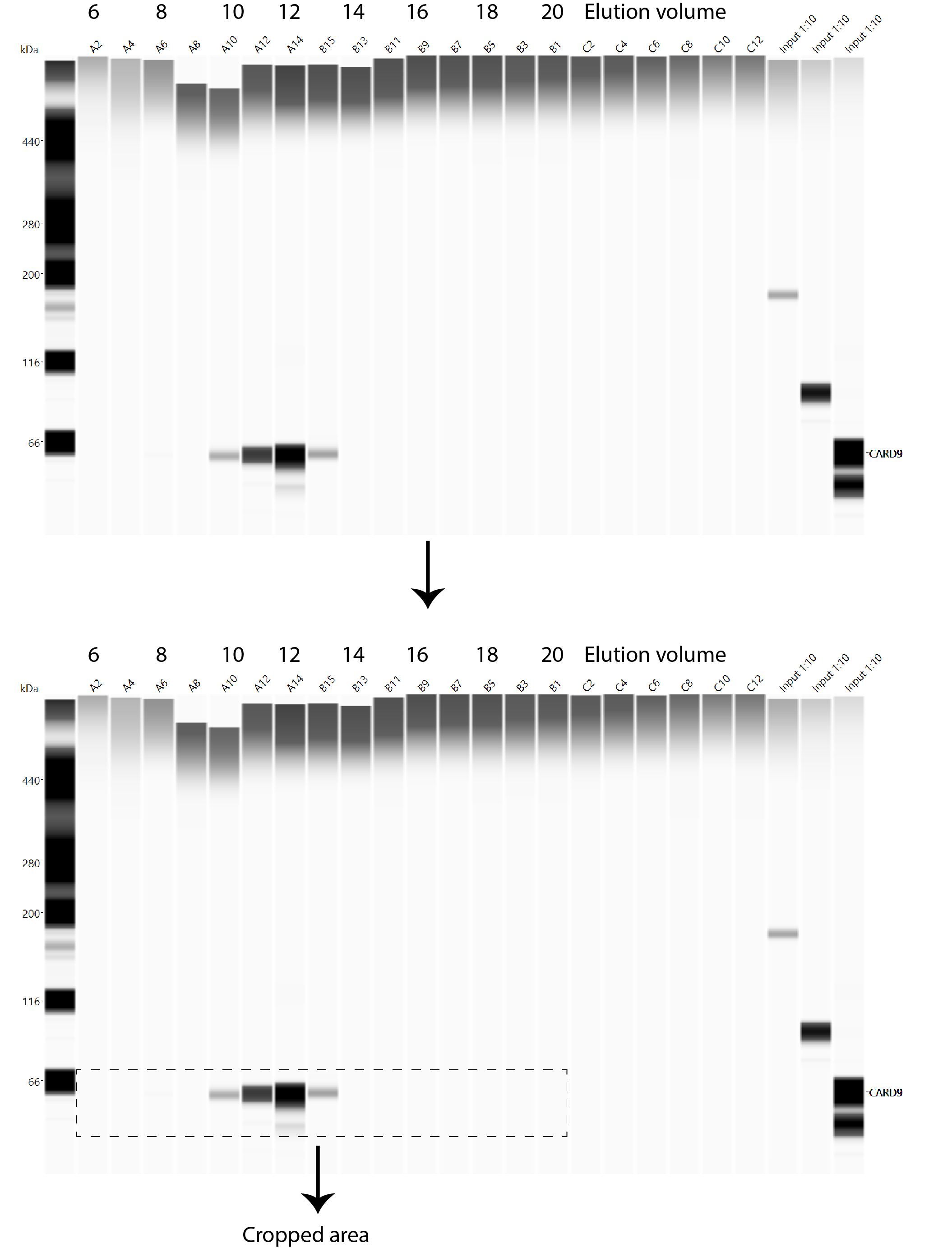

Supplement: Figure 1—source data 2. [file elife-79826-fig1-data2.zip › Figure1-source data 2.png]

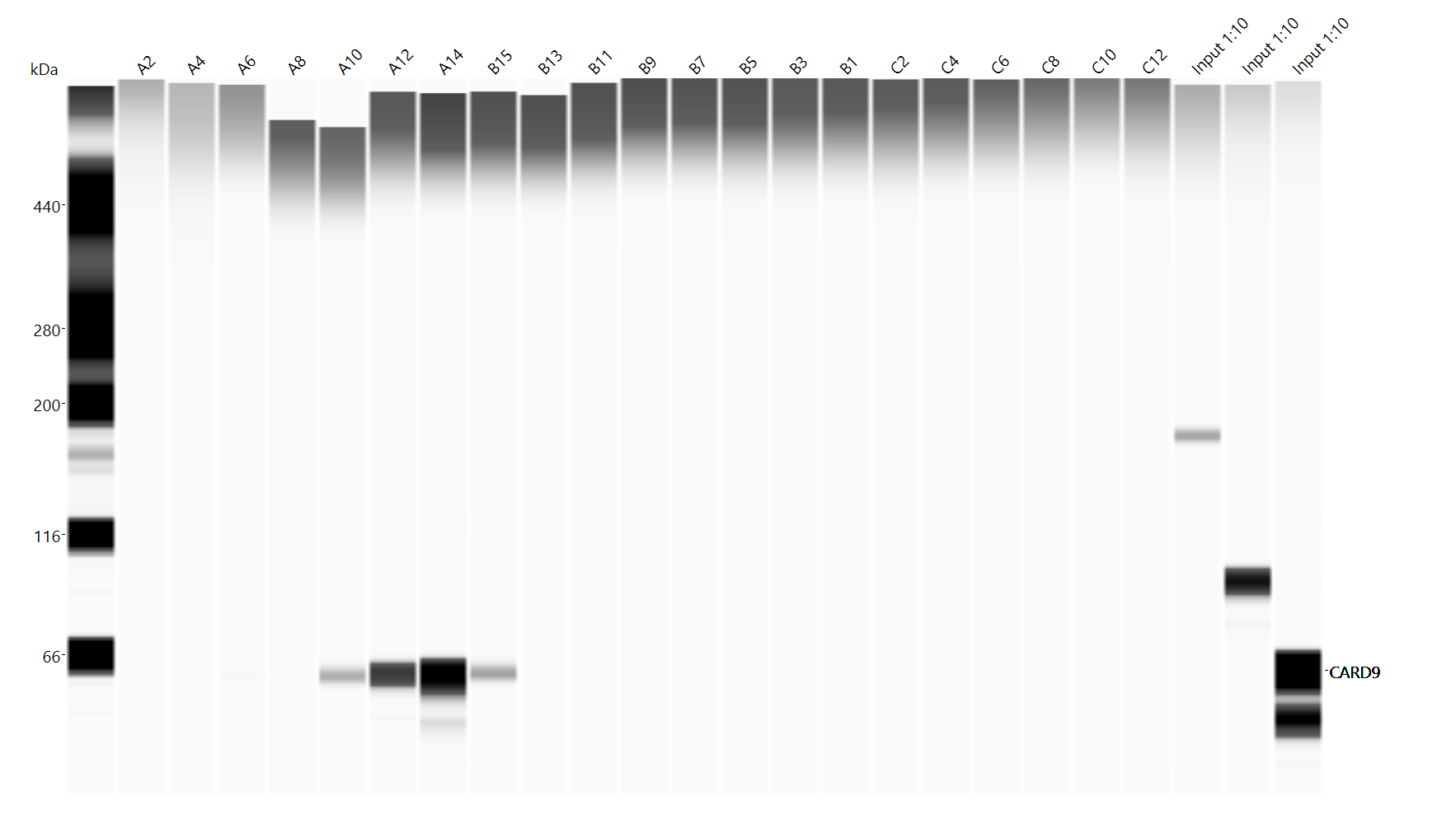

Supplement: Figure 1—source data 2. [file elife-79826-fig1-data2.zip › Figure1-source data 2_raw SEC_THP-1_Ctrl_CARD9.png]

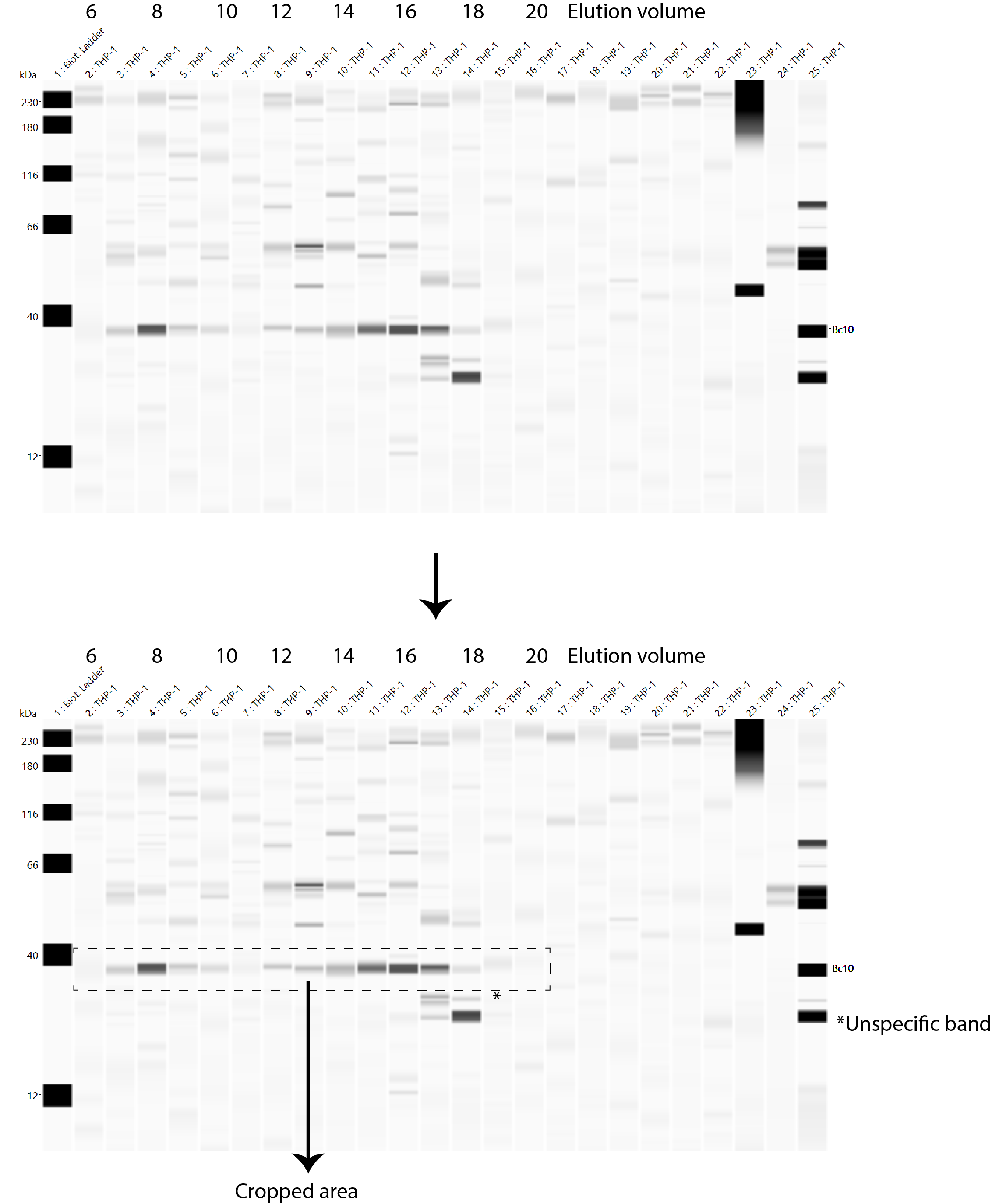

Supplement: Figure 1—source data 3. [file elife-79826-fig1-data3.zip › Figure1-source data 3.png]

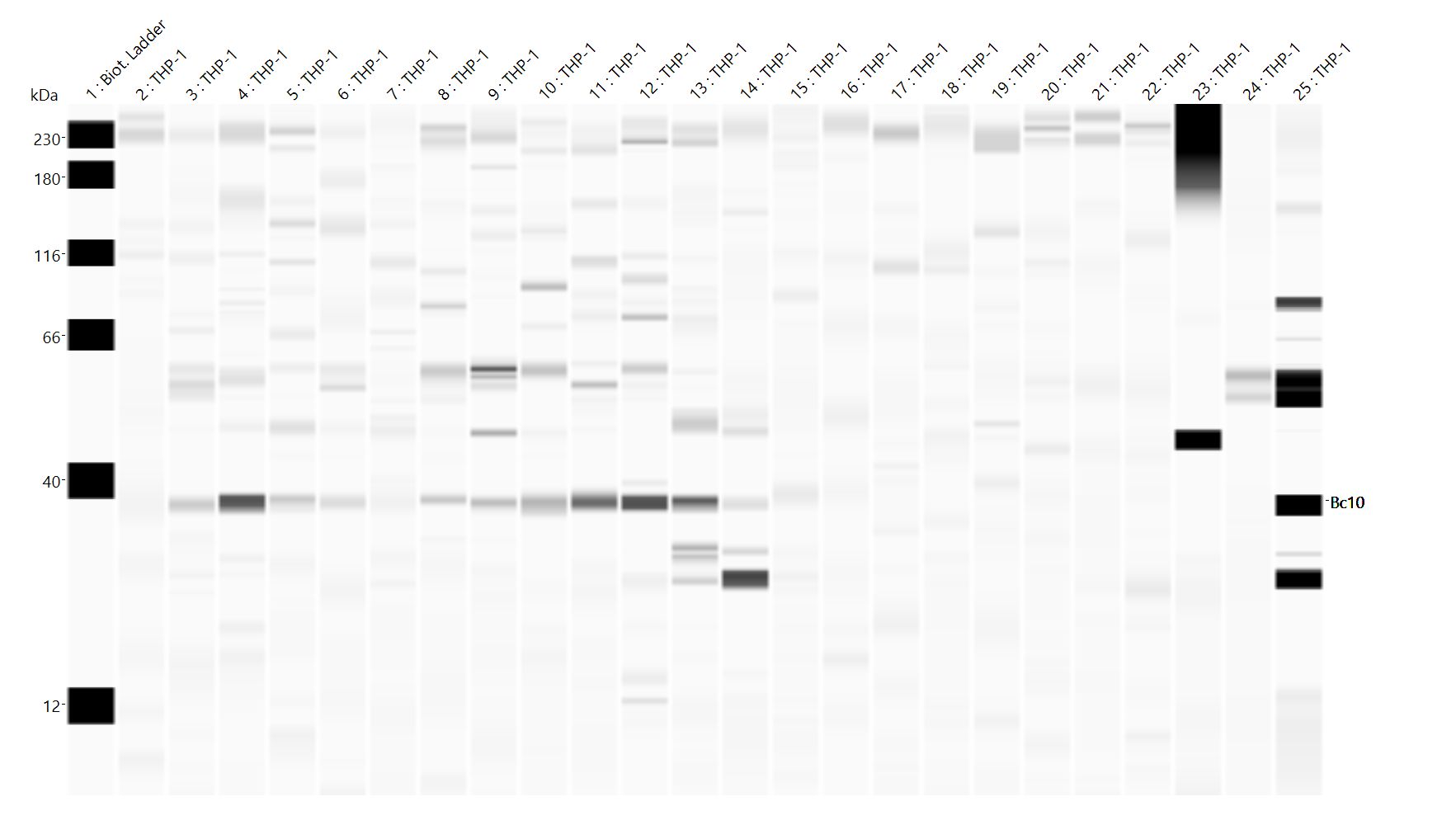

Supplement: Figure 1—source data 3. [file elife-79826-fig1-data3.zip › Figure1-source data 3_raw SEC_THP1_BGP.png]

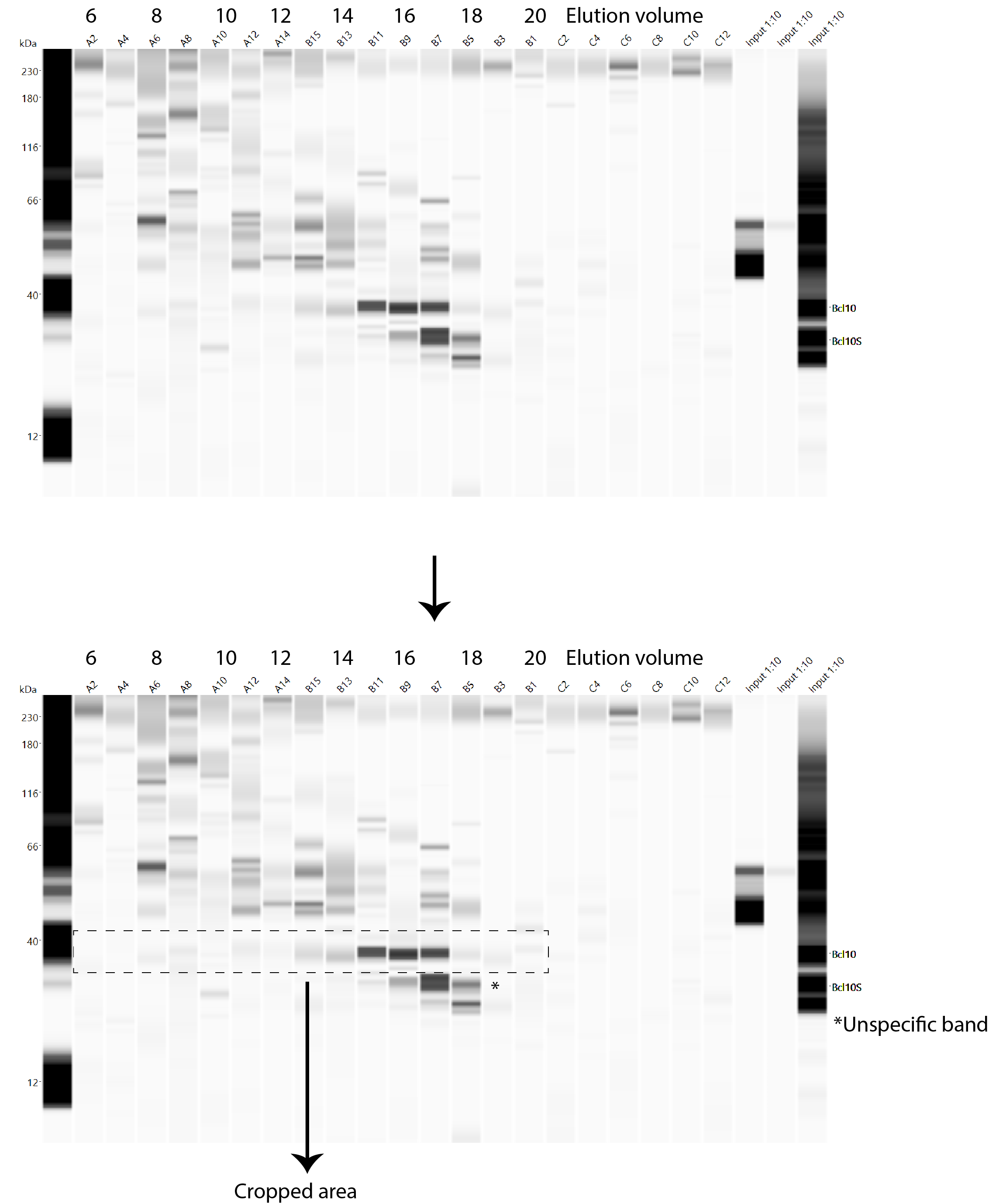

Supplement: Figure 1—source data 4. [file elife-79826-fig1-data4.zip › Figure1-source data 4.png]

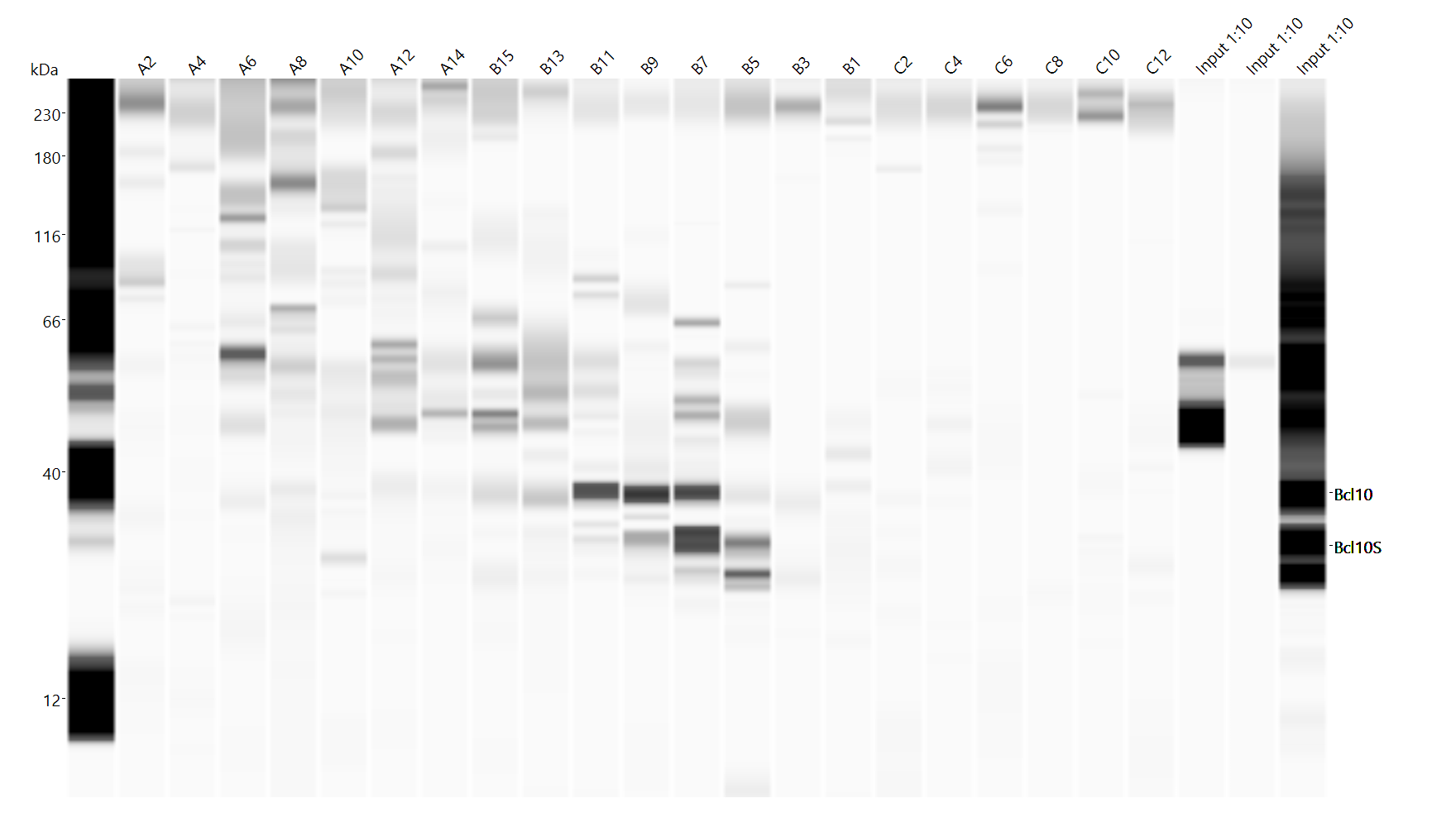

Supplement: Figure 1—source data 4. [file elife-79826-fig1-data4.zip › Figure1-source data 4_raw SEC_Ctrl_Bcl10.png]

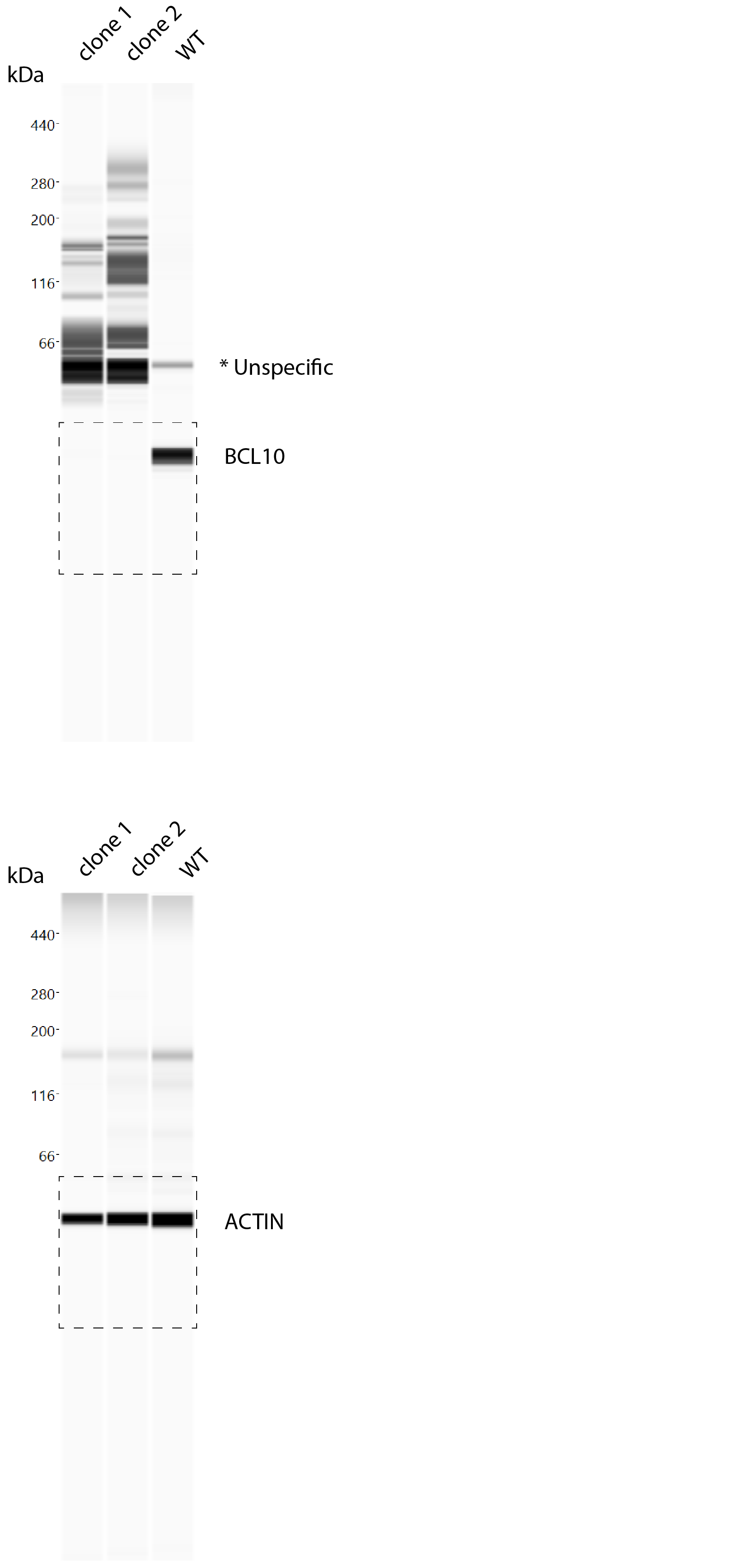

Supplement: Figure 1—figure supplement 2—source data 1. [file elife-79826-fig1-figsupp2-data1.zip › Figure 1-figure supplement 2-source data 1.png]

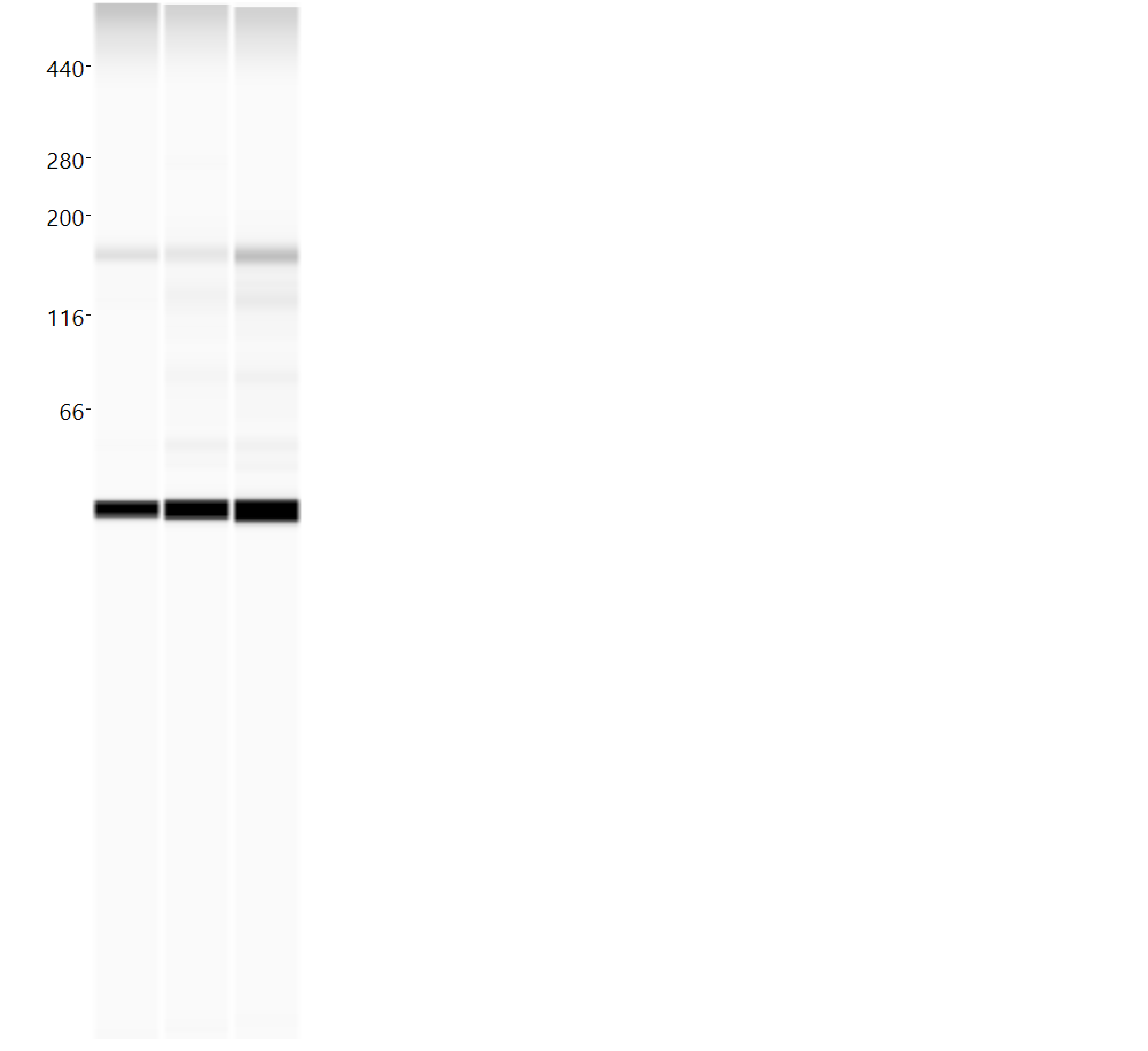

Supplement: Figure 1—figure supplement 2—source data 1. [file elife-79826-fig1-figsupp2-data1.zip › Figure 1-figure supplement 2-source data 1_raw actin.png]

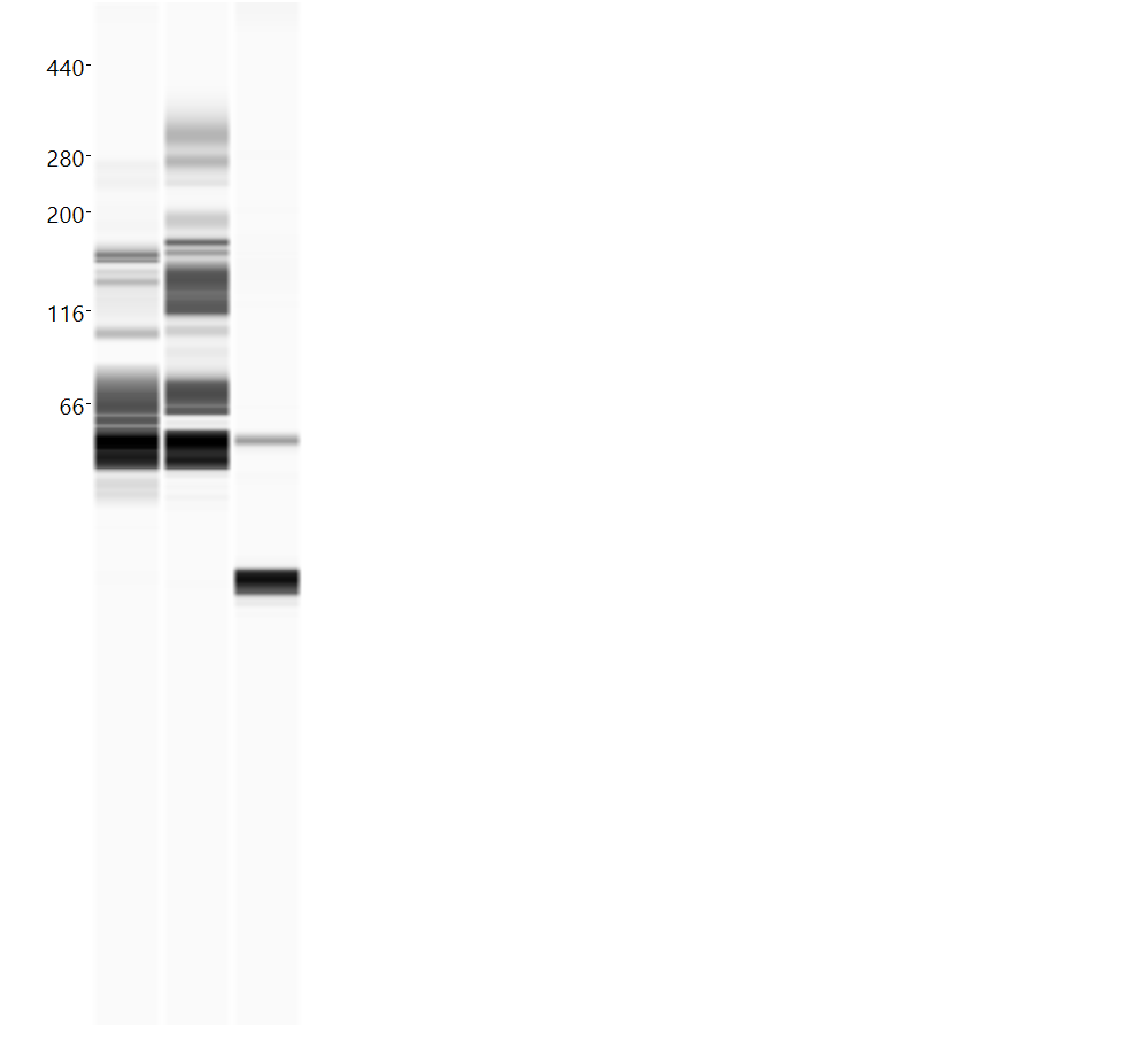

Supplement: Figure 1—figure supplement 2—source data 1. [file elife-79826-fig1-figsupp2-data1.zip › Figure 1-figure supplement 2-source data 1_raw bcl10.png]

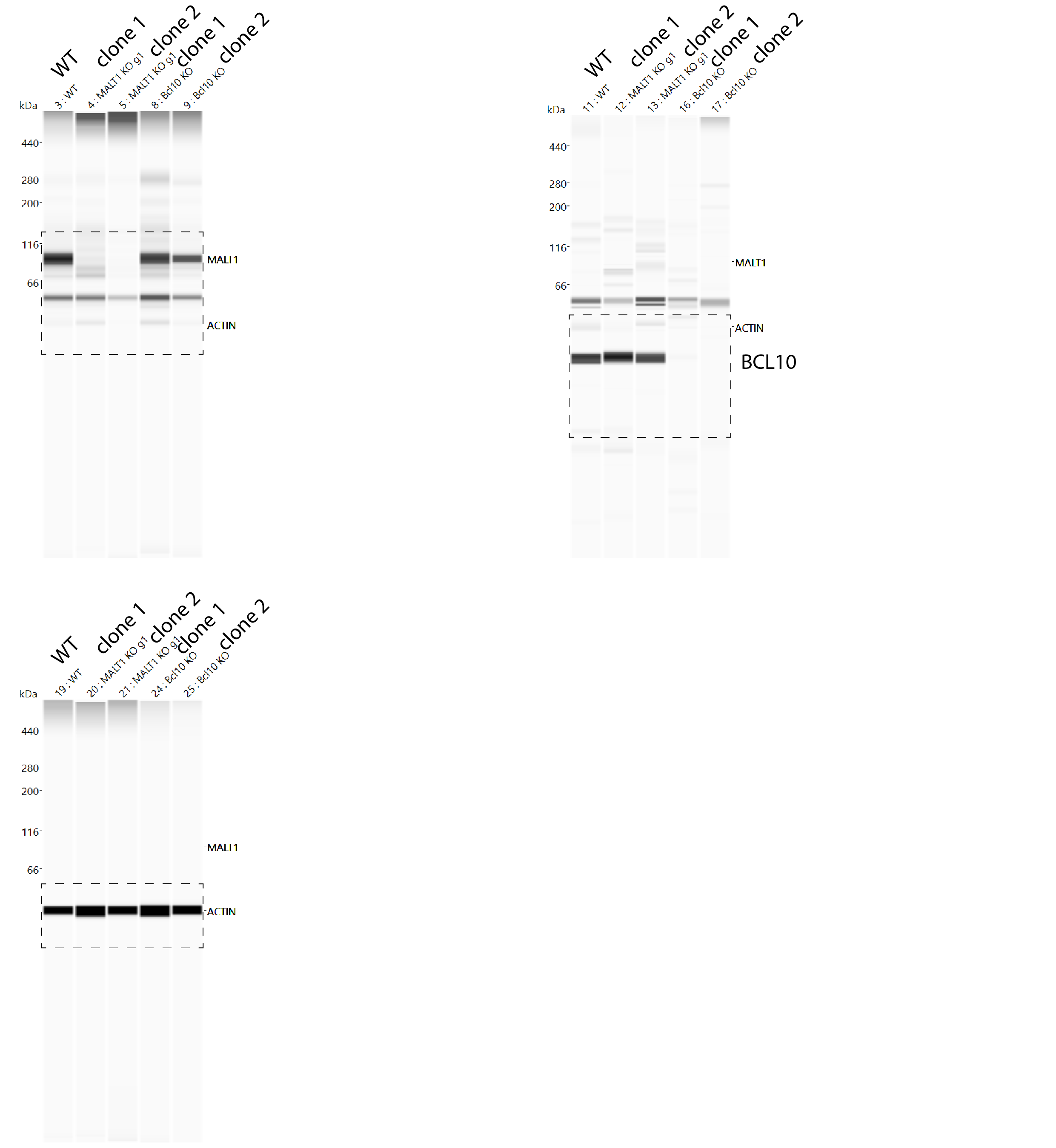

Supplement: Figure 1—figure supplement 2—source data 2. [file elife-79826-fig1-figsupp2-data2.zip › Figure 1-figure supplement 2-source data 2.png]

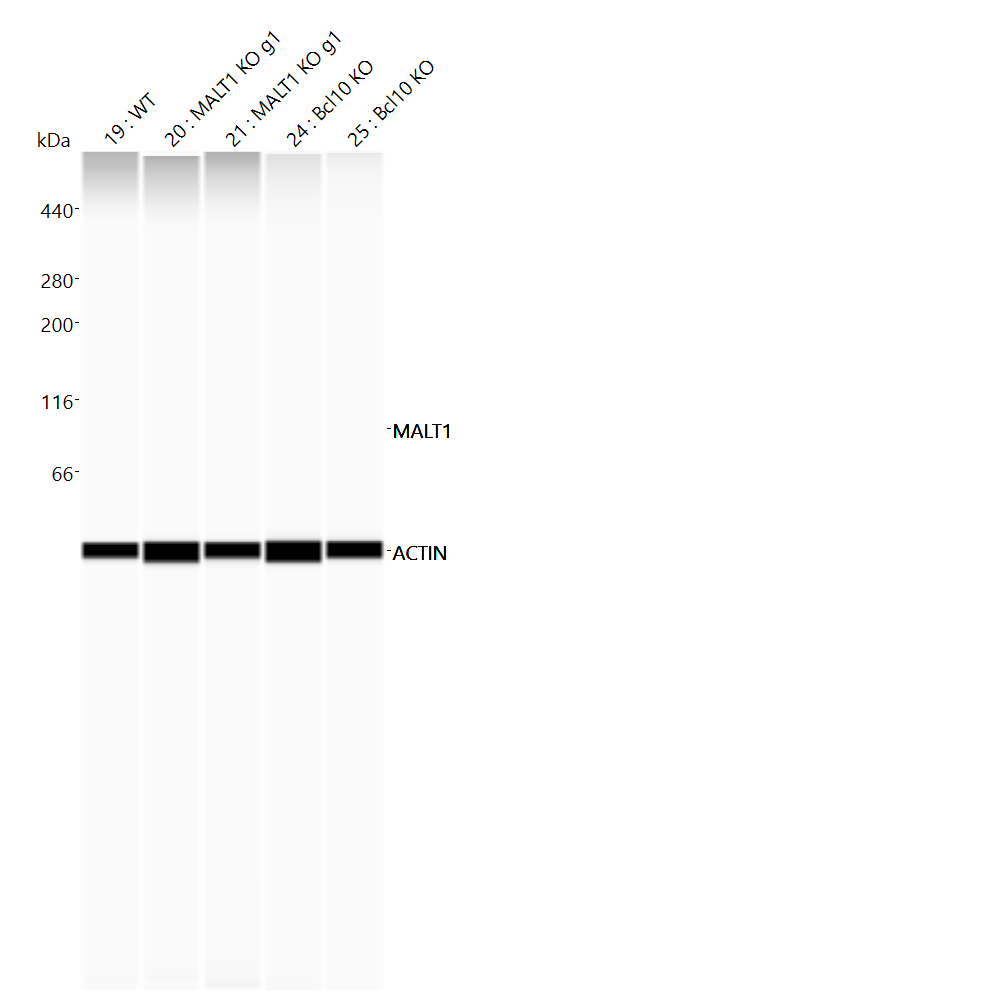

Supplement: Figure 1—figure supplement 2—source data 2. [file elife-79826-fig1-figsupp2-data2.zip › Figure 1-figure supplement 2-source data 2_raw actin.png]

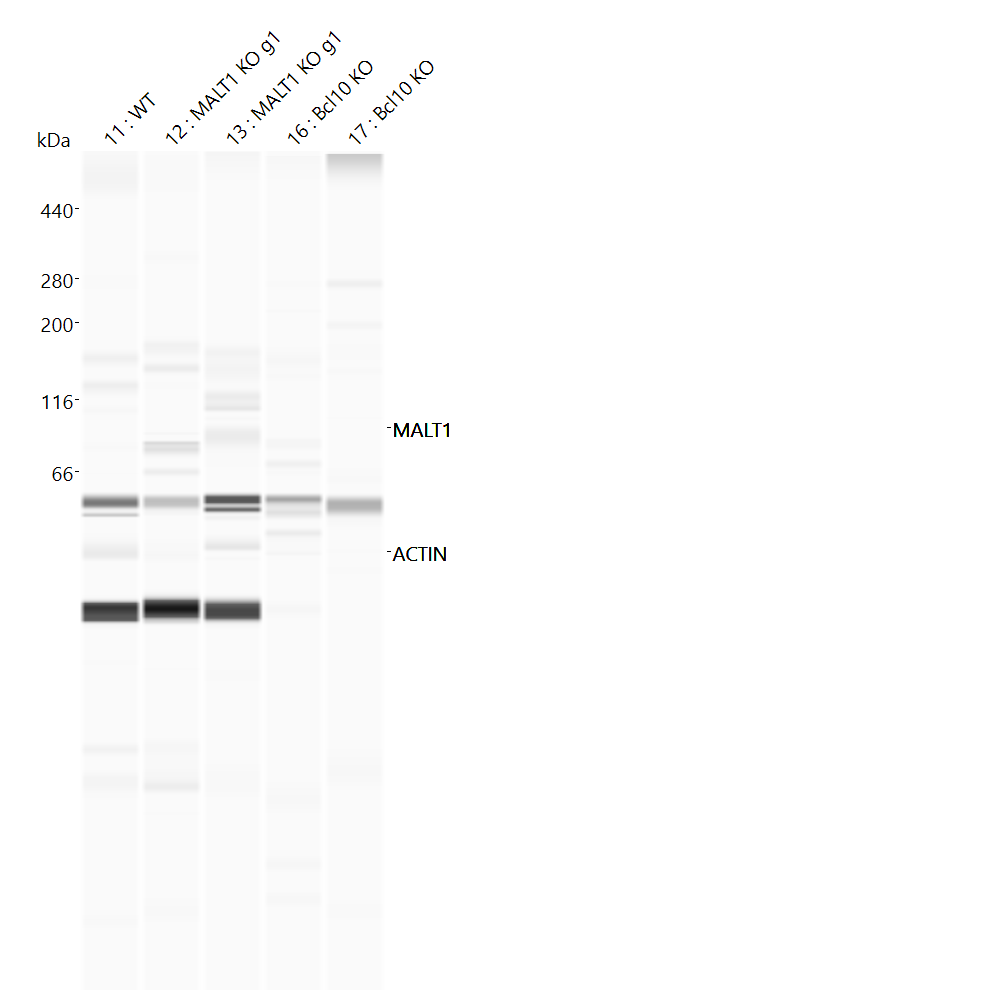

Supplement: Figure 1—figure supplement 2—source data 2. [file elife-79826-fig1-figsupp2-data2.zip › Figure 1-figure supplement 2-source data 2_raw bcl10.png]

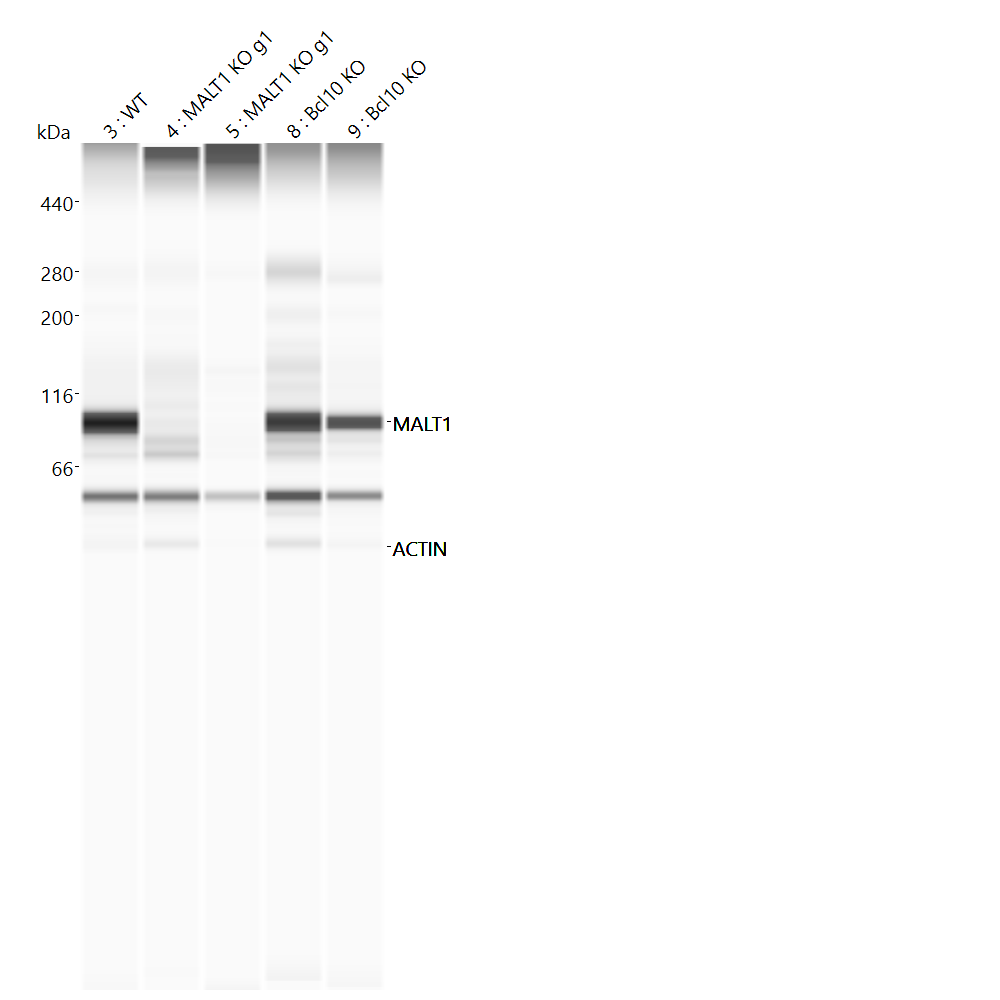

Supplement: Figure 1—figure supplement 2—source data 2. [file elife-79826-fig1-figsupp2-data2.zip › Figure 1-figure supplement 2-source data 2_raw malt1.png]

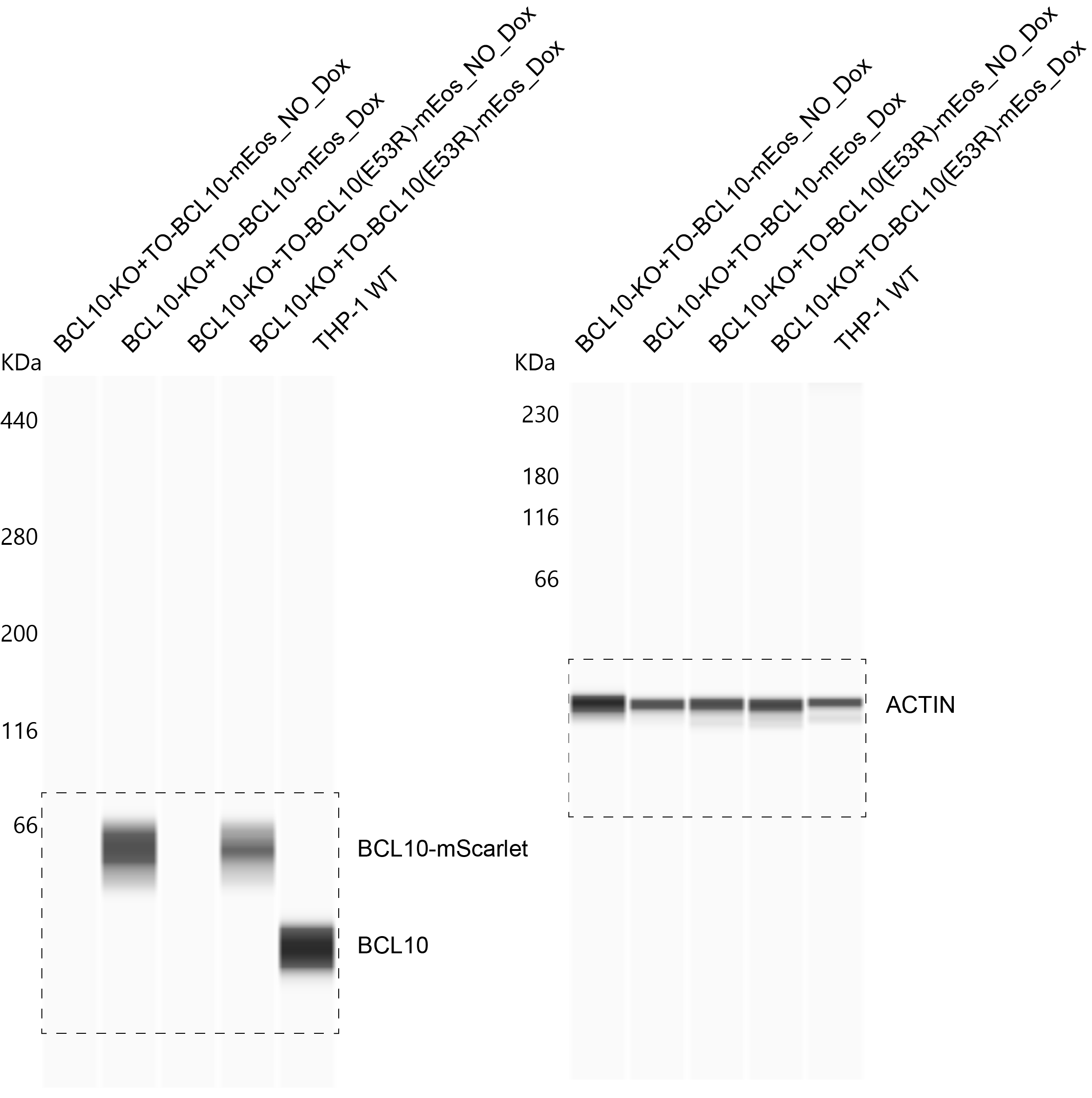

Supplement: Figure 1—figure supplement 2—source data 3. [file elife-79826-fig1-figsupp2-data3.zip › Figure 1-figure supplement 2-source data 3.png]

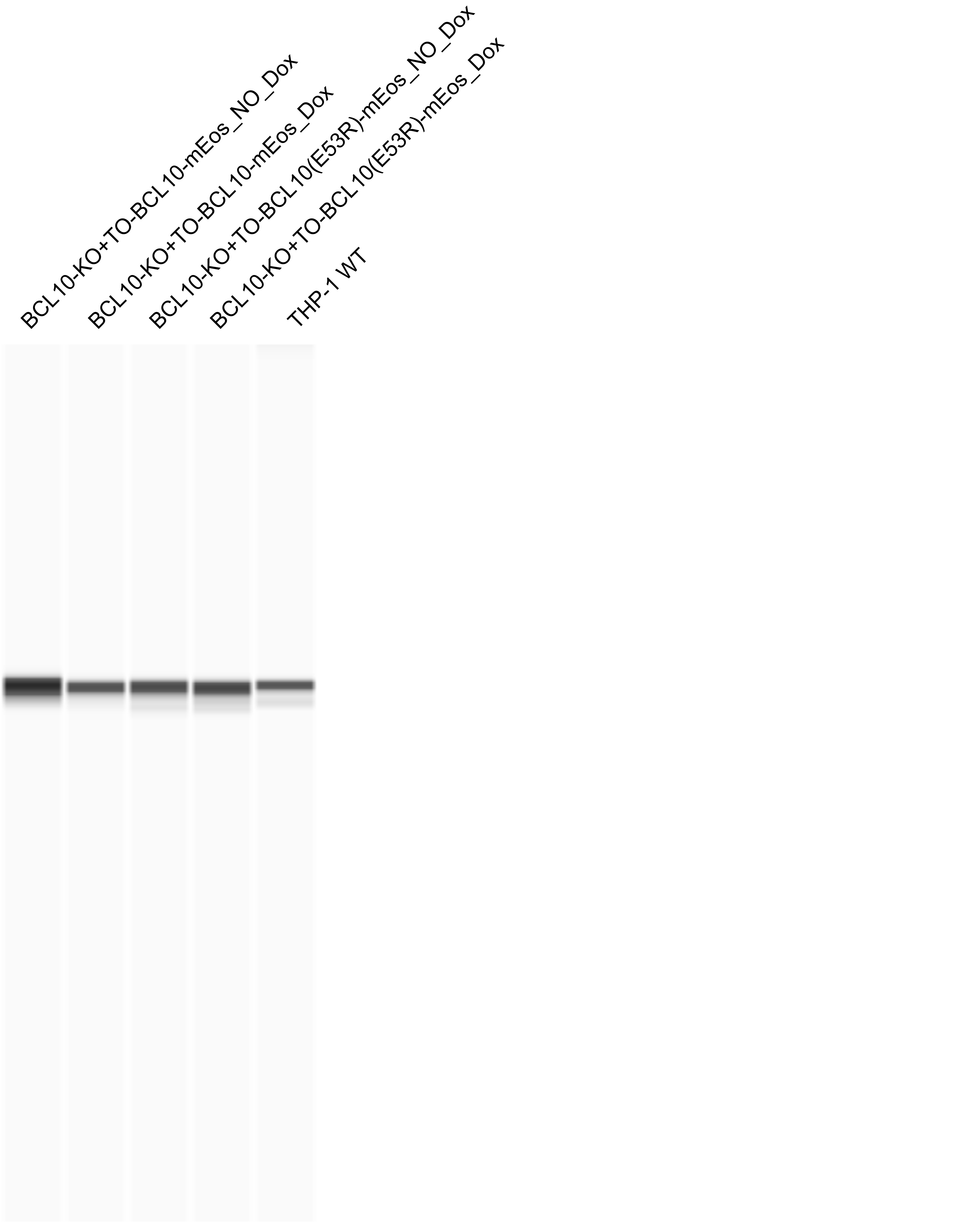

Supplement: Figure 1—figure supplement 2—source data 3. [file elife-79826-fig1-figsupp2-data3.zip › Figure 1-figure supplement 2-source data 3_raw actin.png]

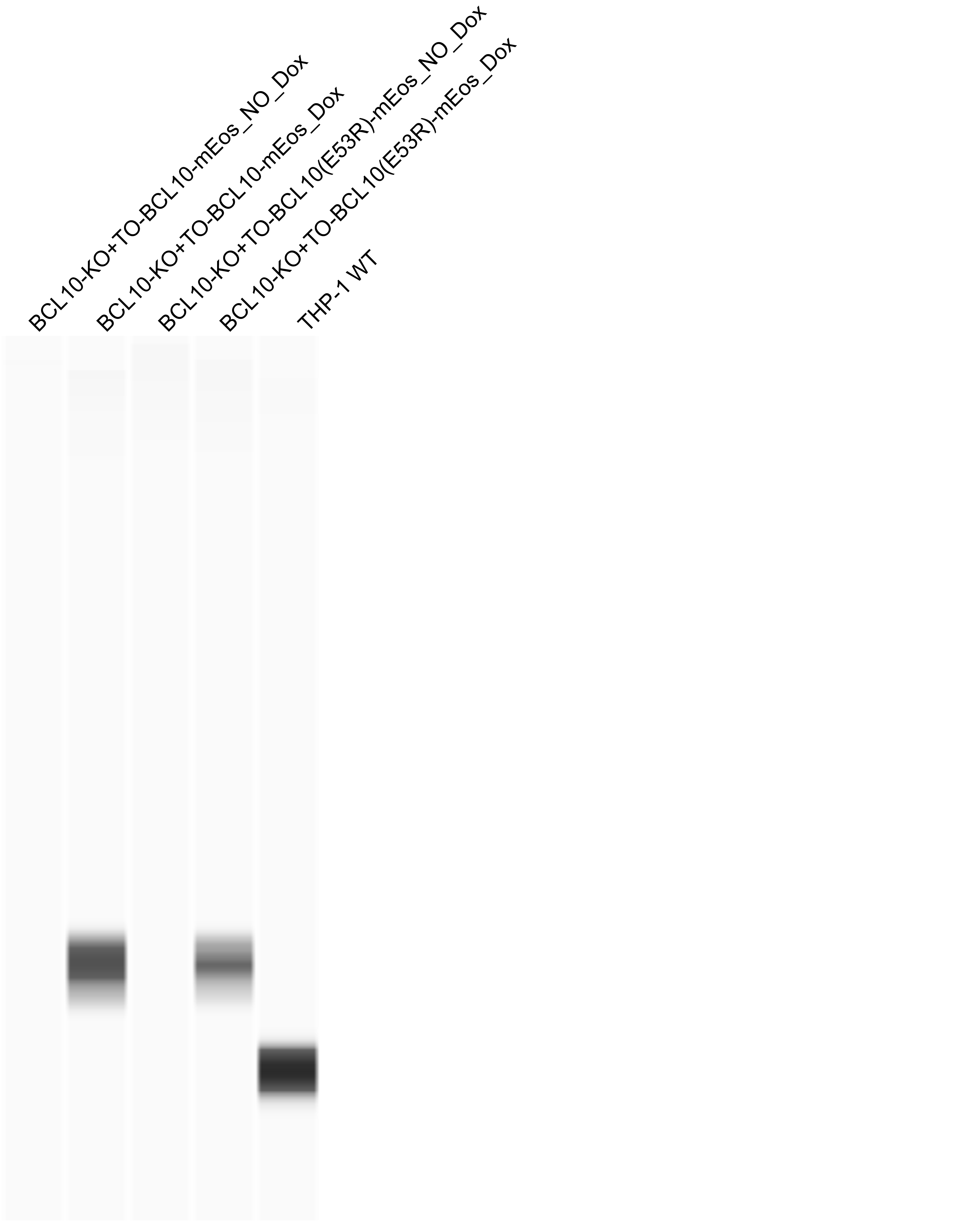

Supplement: Figure 1—figure supplement 2—source data 3. [file elife-79826-fig1-figsupp2-data3.zip › Figure 1-figure supplement 2-source data 3_raw bcl10.png]

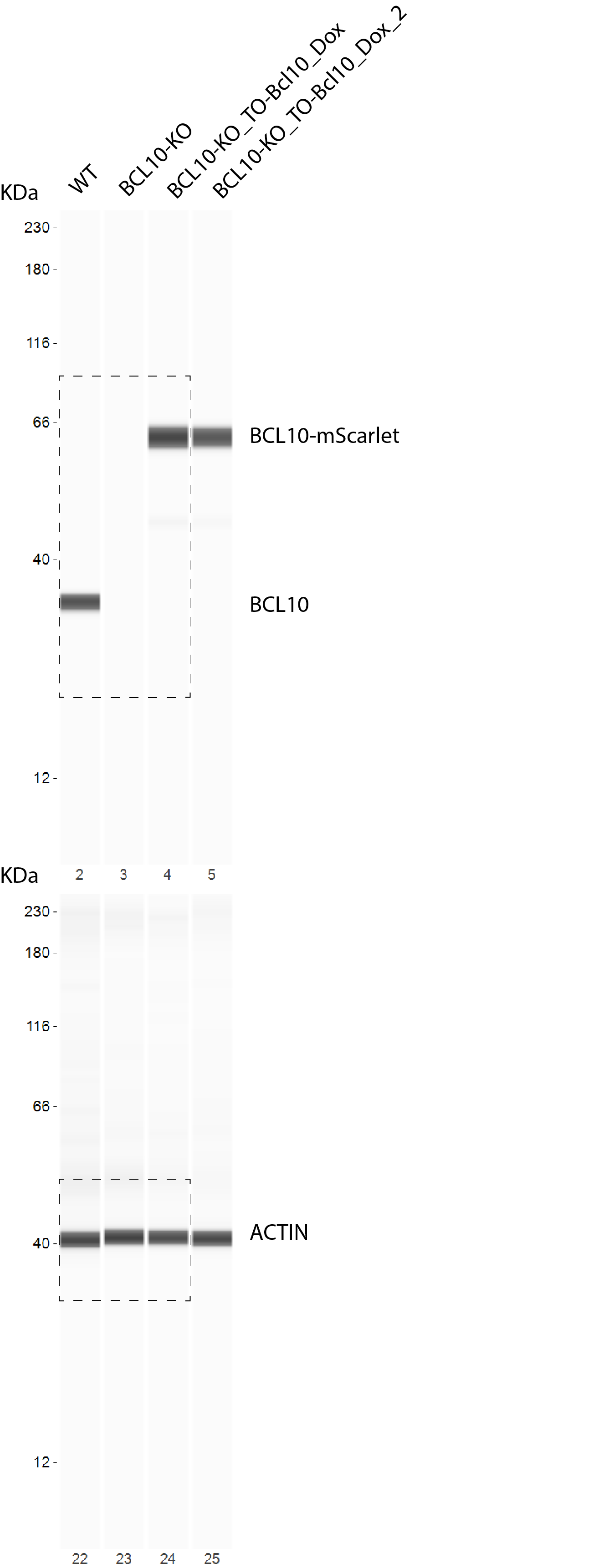

Supplement: Figure 4—figure supplement 3—source data 1. [file elife-79826-fig4-figsupp3-data1.zip › Figure 4-figure supplement 3-source data 1.png]

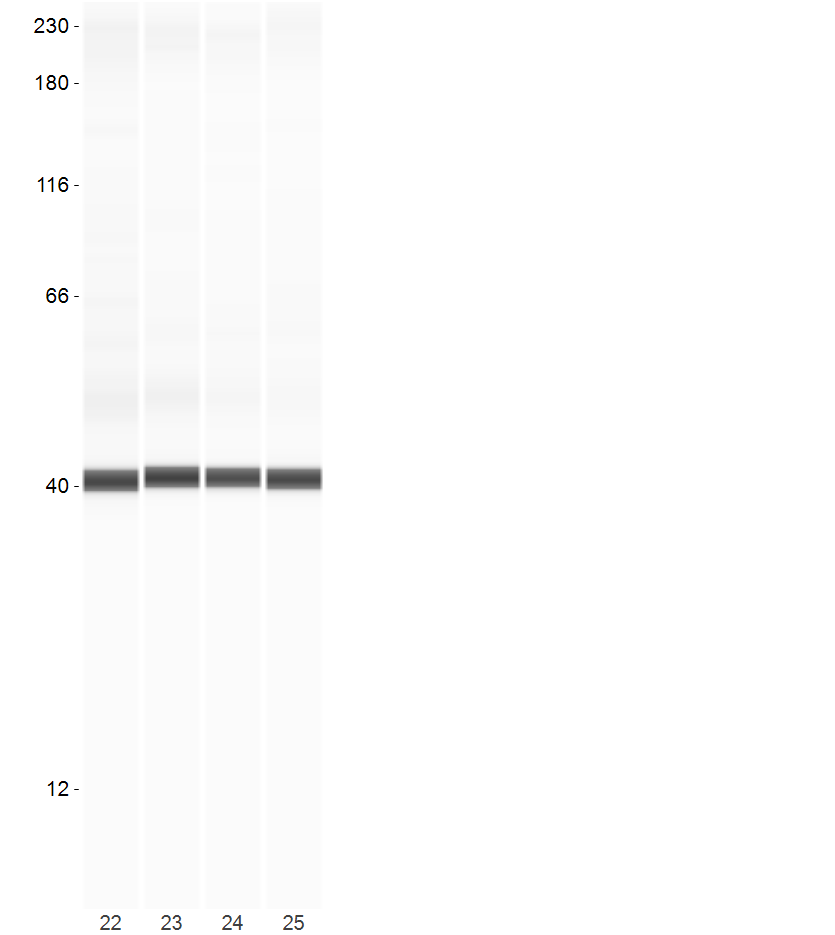

Supplement: Figure 4—figure supplement 3—source data 1. [file elife-79826-fig4-figsupp3-data1.zip › Figure 4-figure supplement 3-source data 1_raw actin.png]

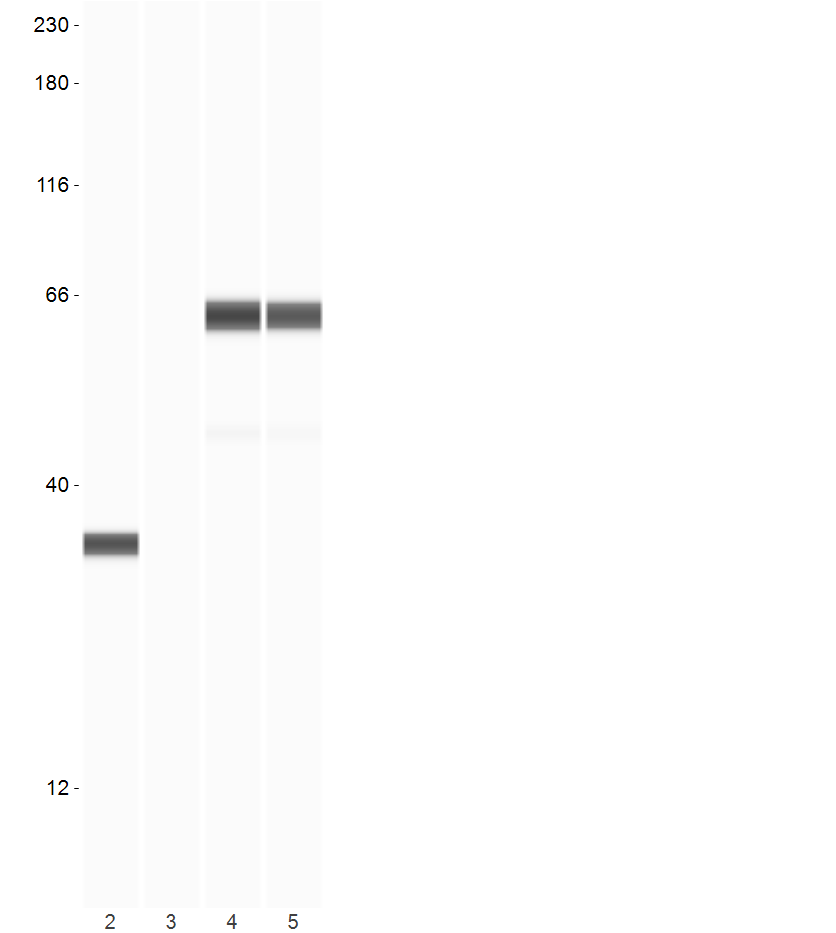

Supplement: Figure 4—figure supplement 3—source data 1. [file elife-79826-fig4-figsupp3-data1.zip › Figure 4-figure supplement 3-source data 1_raw bcl10.png]

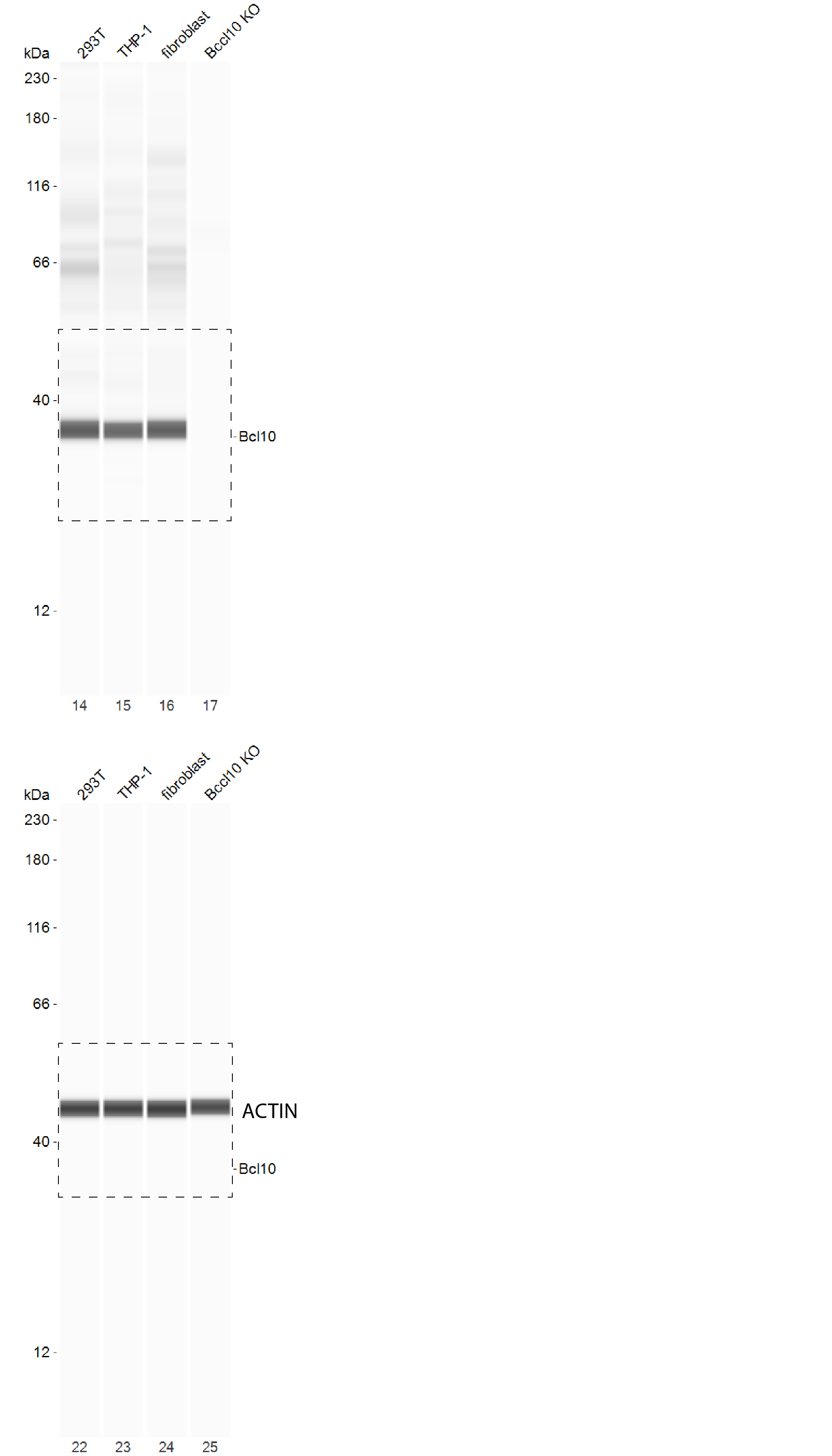

Supplement: Figure 4—figure supplement 3—source data 2. [file elife-79826-fig4-figsupp3-data2.zip › Figure 4-figure supplement 3-source data 2.png]

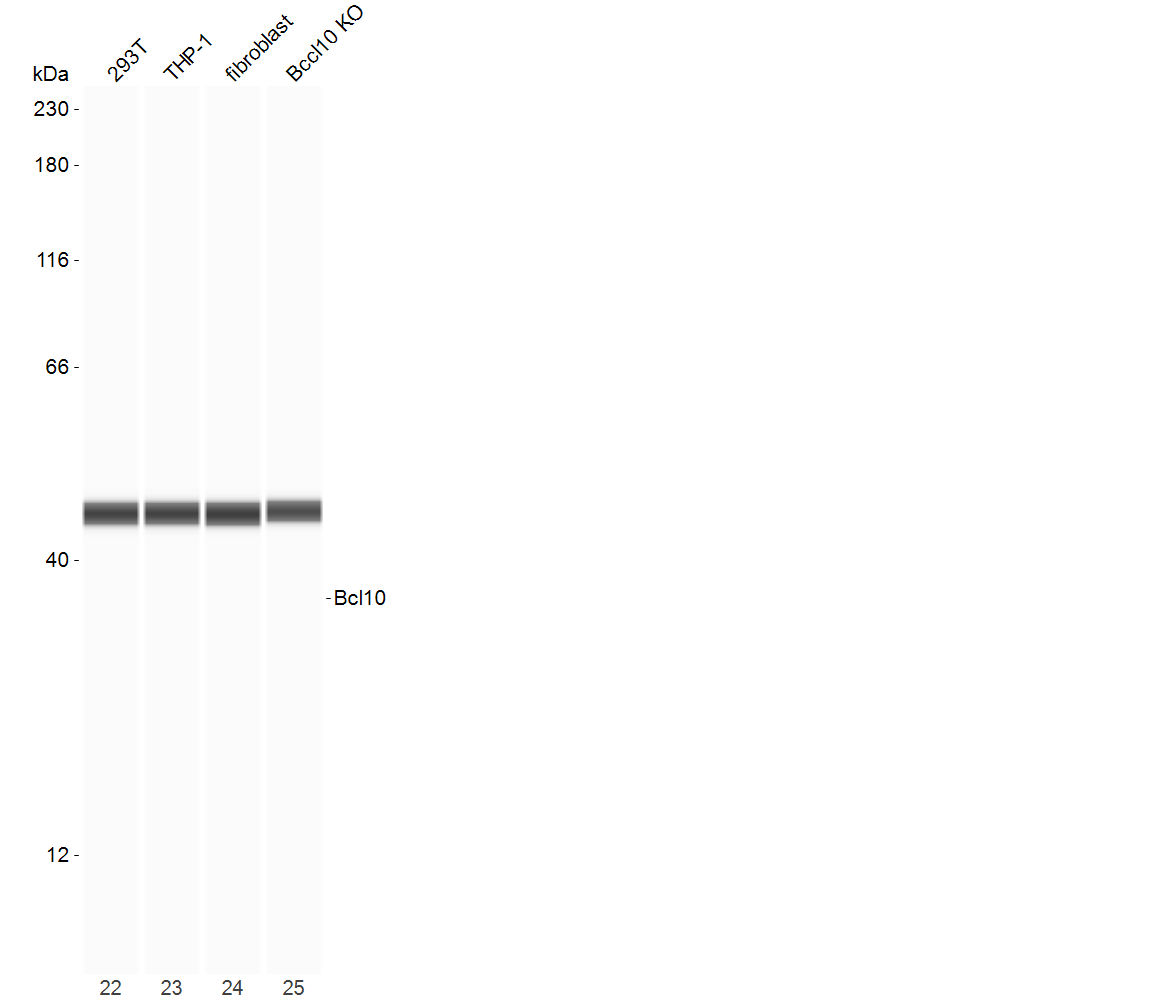

Supplement: Figure 4—figure supplement 3—source data 2. [file elife-79826-fig4-figsupp3-data2.zip › Figure 4-figure supplement 3-source data 2_raw actin.png]

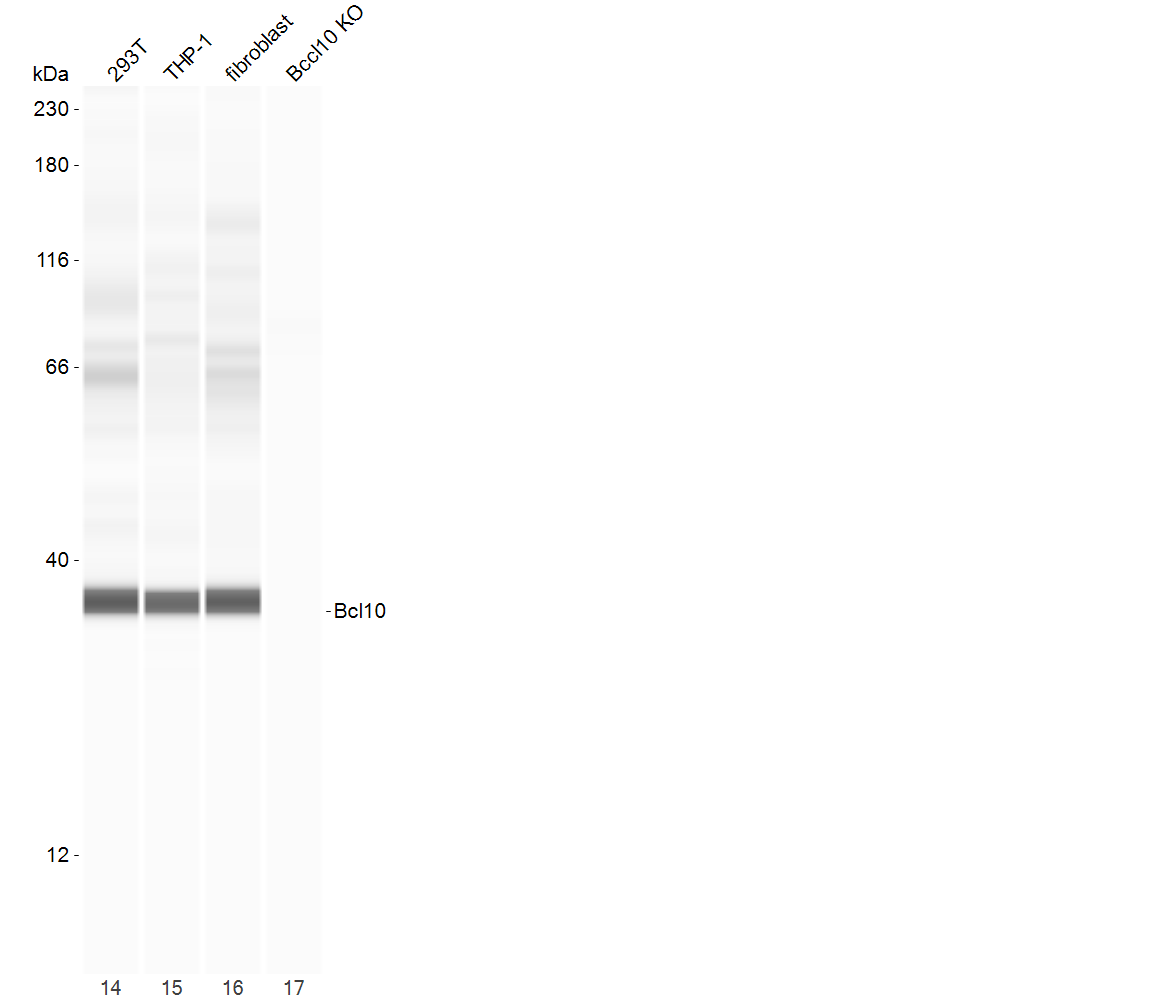

Supplement: Figure 4—figure supplement 3—source data 2. [file elife-79826-fig4-figsupp3-data2.zip › Figure 4-figure supplement 3-source data 2_raw bcl10.png]
